# Kaiy (traditional cautery) in Benghazi, Libya: complications versus effectiveness

**DOI:** 10.11604/pamj.2015.22.98.6399

**Published:** 2015-10-02

**Authors:** Mona Kamal Farid, Abdulla El-Mansoury

**Affiliations:** 1 Department of Pathology, Faculty of Basic Medical Sciences, Libyan International Medical University, Benghazi, Libya; 2 Department of Physiology, Faculty of Basic Medical Sciences, Libyan International Medical University, Benghazi, Libya

**Keywords:** Burning, complications, counter irritant, health care

## Abstract

**Introduction:**

The practice of Kaiy (Cautery) as a traditional therapy is not science based though it is widely practiced worldwide. In Libya, in particular, it is commonly used with no any report or publication to emphasis on its positive or negative impact. This work was undertaken to highlight the complications and disadvantages of kaiy in the Libyan societies as it seems to cause more harm than benefit for the patient.

**Methods:**

We conducted a questionnaire-based survey in the period from the first of March to the end of April (two months) of the year 2013, on fifty patients who were collected from different hospitals in Benghazi city, and who had experienced Kaiy therapy for different diseases.

**Results:**

We found that kaiy application is more common among non educated patients (30 patients, 60%). Most of patients (45 cases, 90%) followed their relatives’ advice and that 32 cases (63.5%) did not improve and show undesirable manifestations and complications.

**Conclusion:**

This study has shown that Kaiy therapy is associated with considerable health risks; therefore, we recommend discouraging and restricting its application.

## Introduction

Kaiy (the Arabic word of cautery) is one of the most ancient medical forms of traditional therapy that is still in use currently. It has been noted that 80% of population from the developing countries use such traditional services either for treatment, prevention of disease and or maintenance of good health [1]. The ancient Egyptians had great faith in the therapeutic values of fire so they used cautery to stop bleeding [2]. One of the people recognized in the treatment methods using cauterization is the great Andalusian physician, Abu al-Qasim al-Zahrawi, who lived in the 10th century [3]. He developed special tools called cauters for use to stop bleeding of arteries. The instrument used nowadays is usually a metal rod that is pointed at one end or bent at the top into a crescent shape. Different parts of the body are cauterized for different ailments and diseases [4]. Cauterization involves creating burns on the tissue to either close wounds or stop bleeding because the heat would make the blood clot, or to remove part of the body [3]. The number of cautery in one session varies between one to seven and more. The choice of the location for the application of cautery depends on the patient's complaints [3]. For example in cases of jaundice, the cautery is applied to the left hand; while for sciatica, it could reach up to 17 cautery buns at different locations. If the patient complains of chest pain with shortness of breath, which could be angina or myocardial infarction, it is applied to the 4th and 5th anterior or posterior ribs on the same side of the pain [3]. In the diabetic foot, it is commonly applied to the dorsum of the foot or the lateral aspect of the lower leg [5].

“Is cautery useful, or are we just burning people?” [2]. Cautery was thought to prevent infection, but recent research has proven otherwise. In addition, there was a belief that intense heat destroys the pathogenic substances inside the body [6]. There is another thought that the formation of blisters is essential if healing is to occur [3]. Treatment by kaiy is a crude method of applying a counter irritant [1]. Patients, who have received traditional cautery treatment, report that it gives temporary relief of their symptoms followed by severe pain. Kaiy may possibly act in the same way as acupuncture, stimulating the release of endogenous opioids and other neurotransmitters that prevent the feeling of pain that is a natural physiological body method to prevent the feeling of continuous pain [2]. But how do we burn patients making them suffering more pain to treat pain. Pain for pain is non-sense [2]. Expected complications due to delayed medical presentation are commonly observed. These include deep skin burns, wound infection, and delayed wound healing leading to amputation, septic shock, tetanus and multiple abscesses [4]. In addition, when kaiy is applied as a cure resort of critical cases, especially cancer, delayed management would increase the aggressiveness of the disease that makes it difficult to be controlled hence have deleterious effects on patient's health [7].

## Methods

We collected fifty cases that had experienced kaiy. These cases were collected randomly from different hospitals in Benghazi- Libya. Patients were given a questionnaire to be fulfilled by themselves that includes different related points as their level of education, history of their complaints, the reason of using traditional cautery, etc,. An official letter was written to the hospital manager for his agreement. The nature of the study was explained to all participants and we got their verbal and written consent before participation.

## Results

Fifty patients who had experienced kaiy were collected randomly from different hospitals in Benghazi during a period of two months, from the first of March to the end of April of the year 2013 and a related questionnaire was fulfilled by them. The obtained results were as follows

### Education level

Thirty patients were non educated (60%), while 20 patients were educated (40%) with education level ranges from moderate to high.

### Advisers to apply cautery

Forty five patients (90%) followed their parents’ or relatives’ advice and only 5 patients (10%) did not have a family history of previous cautery application.

### The reason for experiencing cautery

Patients experienced traditional cautery because they wanted a rapid improvement of their complaints regardless of the applied technique either painful or harmful. They also mentioned that medical treatment takes too long time before noticing beneficial effects and sometimes it fails or needs the performance of many investigations before its application.

### The reason for seeking medical advice

Patients sought medical advice because they noticed no improvement or after feeling worse or developing complications.

### The effect of Kaiy on the patients’ complaints

**Figure 1:**
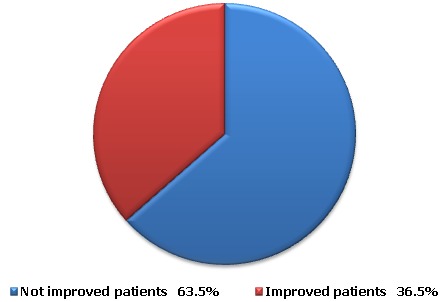
The effect of Kaiy on complaints of the patients

**Figure 2:**
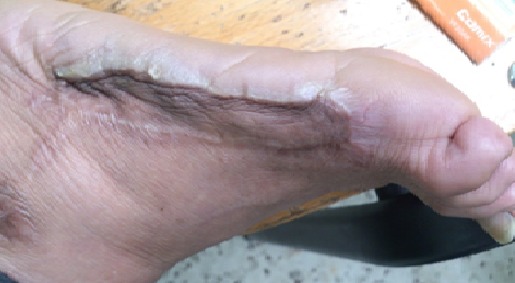
Keloid and disfigurement along the lateral aspect of the left foot skin

**Figure 3:**
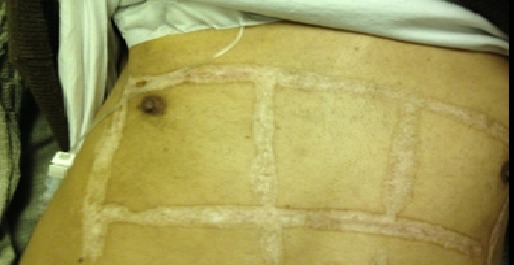
Multiple scars with disfigurement of the chest skin

**Figure 4:**
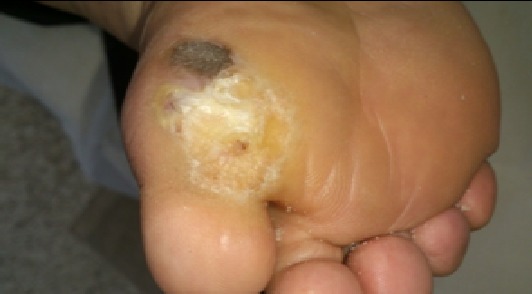
Infected deep seated burn with infected eroded overlying skin

**Figure 5:**
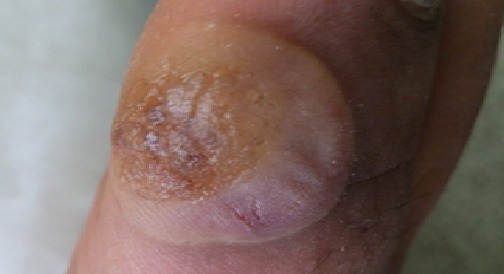
Infected blister

Out of the total collected 50 cases, 18 patients (36.5%) were improved. On the other hand, 32 patients (63.5%) not only did not get any benefit from the traditional cautery application but also had undesirable manifestations and complications (Figure 1). The undesirable manifestations were mainly in the form of disfigurement and keloid (Figure 2, Figure 3). The complications varied from infected deep-seated burns (Figure 4), infected blisters (Figure 5), multiple abscesses, severe bleeding followed by coma to septic shock or deterioration of the condition with loss of body weight and appetite due to delayed receiving the appropriate treatment. Some of the complicated cases were diabetics, started their treatment firstly by Kaiy. Then they noticed a gradual decreased sensation in their legs. Their condition was deteriorated to the degree of total loss of sensation and darkening of the over lining leg's skin. These cases of dry gangrenous diabetic foot will be treated by surgical amputation. One patient had ophthalmalgia and was exposed to Kaiy for one year and half without any improvement. This condition ended by blindness and atrophy of the eye ball. Another patient suffered from chronic disturbance of bowel habit for two years. He experienced traditional cautery but did not benefit of it. On the other hand, he started losing weight and appetite. On seeking medical advice and by investigations, it proved to be an advancing stage of colonic cancer. Two old women had a history of irregular vaginal bleeding. They went to traditional healers claiming improvement by Kaiy, but their bleeding became more severe. They became weak, pale and additional complaints of constipation and blood in stool started to appear. By medical examination and after performing different investigations, these cases were proved to be advanced late stage of uterine cancer spreading to the adherent loops of bowel and liver. Now radical hysterectomy and chemotherapy will be performed.

Another old aged patient had a history of hemoptysis for one year. He had been cauterized on his head and behind the ears for six months. When he noticed no improvement, he was admitted to the hospital and was diagnosed as lung cancer. Now he is on chemotherapy treatment. A diagnosed breast cancer female patient, instead of performing simple mastectomy as was advised by physicians, preferred a traditional cautery because she had fear of surgery. The cautery was applied to the head region and over the breast mass. She was informed by the healer to avoid wetting the burnt area till it becomes infected - as pus formation lets the causative agents to be discarded from the body and hence cure, according to the healer's belief- Following his instructions, the affected area became infected and started to discharge greenish yellow suppurative fluid with persistence of the breast mass that showed an increase in size with enlargement of the axillary lymph nodes. Now the patient is hospitalized to perform radical mastectomy with axillary clearance.

## Discussion

Many patients in developing countries use traditional therapies before seeking medical advice; it is a common belief since ancient times, that Kaiy is the treatment of choice or the first resort to many diseases. It has been noticed that parents are the main influence on seeking cautery treatment and the patients claiming traditional cautery are of low education level if not uneducated [8]. In our study, 45 patients (90%) followed the advice of their parents or relatives and only 20 patients (40%) were educated. It has been reported that a very common reason for the delayed medical presentation, even among the educated patients, is the phobia of undergoing surgery particularly in cases that require radical excision [8]. People believe in our part of the world that they should not loss any part of their body even if this leads to death [8]. In our study, 32 cases (63.5%) showed complications and undesirable manifestations and only 18 cases got benefit of Kaiy. It has been mentioned that the majority of the reported cases lacked the therapeutic benefit of traditional cautery and they developed complications^1^, [2, 5, 8]. Many traditional medicinal practitioners are people without education, who have rather received knowledge of medicinal plants and their effects on the human body from their forebears. Some healers learn the trade through personal experience while being treated as a patient who decides to become healers upon recovery. Another route is received the knowledge and skills passed down informally from a close family member such as a father or uncle, or even a mother or aunt in the case of midwives [9]. Traditional healing practices reflect the culture and beliefs of a society. Traditional healers are the most easily accessible health resource available to the community. The reason for the continued use of traditional healers is that traditional healers are participants in the culture, they can be found almost everywhere at any time. Most, if not all traditional healers, have not received any form of professional training or licensing [9]. In our study, 18 patients (32.5%) were improved and got benefit of traditional cautery. It has been found that the success of traditional cautery in treating many acute and chronic ailments was the reason behind it is being chosen as the first choice for treatment as for conjunctivitis, headache, migraine, ear infections, tuberculosis, bone fractures, epilepsy, psychosomatic troubles, depression, diarrhea and to stop bleeding; despite lack of scientific data supporting its efficacy or safety [6, 10, 11]. In addition, it was stated that if a patient tried traditional cautery first and got cured, the repercussion would be that most of the patients will try traditional cautery first and will take recourse to the hospitals at a late stage of the disease that clinically means that the condition is becoming worse [5].

## Conclusion

Lack of evidence-based scientific data on Kaiy safety or efficacy does not deter people in Libya from flocking the traditional healers; this may be due to the lack of awareness and education of patients, a delayed medical presentation of their problem till a late stage when serious complications, amputation, radical operations or even death will be the end result. Kaiy as a traditional therapy should be discouraged as it seems to cause more harm than benefit for the patient. Health care systems, health care providers and religious leaders in the society should move towards convincing the public and restricting this undesirable practice. Health authorities should also use multi-media health education to advise of its dangerous outcomes and to change public opinion and belief. Control of local healers will also be of great value.
